# Exploring the Underlying Mechanisms of Reduced Elasticity in PA6/PA66 Bicomponent Melt-Spun Fibers: An Investigation of Viscoelastic Properties and Simulation Analysis

**DOI:** 10.3390/polym17172312

**Published:** 2025-08-27

**Authors:** Ali Abbas, Shengming Zhang, Huaping Wang, Jing Wu, Peng Ji, Chaosheng Wang

**Affiliations:** State Key Laboratory for Modification of Chemical Fibers and Polymer Materials, College of Materials Science and Engineering, Donghua University, Shanghai 201620, China; 417006@mail.dhu.edu.cn (A.A.);

**Keywords:** PA6/PA66 bicomponent fibers, melt spinning simulation, viscoelastic properties, crystallization kinetics, fiber elasticity, finite element modeling, Phan–Thien–Tanner model, process optimization

## Abstract

This study conducts a detailed viscoelastic simulation of the side-by-side PA6/PA66 bicomponent melt spinning process to investigate the mechanisms behind reduced fiber elasticity. A two-dimensional (2D) axisymmetric finite element model was developed using ANSYS Polyflow, incorporating the Phan–Thien–Tanner (PTT) constitutive equation and a non-isothermal crystallization model. Simulation outcomes were validated with experimental and published data, showing close agreement in fiber radius, velocity, and temperature profiles (within 8% deviation). Results indicate that the dominance of the higher-viscosity PA66 phase induces uneven stress distributions and localized crystallization, leading to decreased elastic recovery. Higher winding speeds amplify this effect. This work offers a predictive framework for optimizing industrial melt spinning conditions to improve elasticity in bicomponent fibers. Key results indicate that the dominance of the PA66 component—due to its higher melt viscosity—leads to uneven stress distribution, elevated tensile stress, and localized crystallinity peaks along the spin line. These factors collectively contribute to reduced elastic recovery in the fiber. Moreover, increased winding speeds amplify axial stress and crystallinity disparities, further exacerbating the stiffness of the final product. In contrast, better elasticity was associated with lower pressure drop, balanced crystallinity, and minimized axial velocity differences between the two polymer phases. The findings offer valuable insights into optimizing industrial melt spinning processes to enhance fiber elasticity. This research not only improves fundamental understanding of viscoelastic flow behavior in bicomponent spinning but also provides a predictive framework for tailoring mechanical properties of fibers through process and material parameter adjustments.

## 1. Introduction

High-performance bicomponent fibers are critical in applications ranging from textiles to composites due to their tunable mechanical and thermal properties. Among these, side-by-side PA6/PA66 configurations offer unique potential in enhancing elasticity and strength.

Traditional melt spinning simulations have employed 1D and 2D models to study flow dynamics and fiber formation. Earlier works by Henson et al. [1], Fisher et al. [2], and Keunings et al. [3] focused on isothermal or simplified assumptions. More recent studies (e.g., Sun et al., 2020 [4]; Kim et al., 2023 [5]; Zhang et al., 2021 [6]; integrated crystallization kinetics and viscoelastic modeling to better capture the behavior of polymer blends during spinning.

However, most prior models do not fully explore how mismatched viscoelastic properties of PA6 and PA66 affect the elastic recovery in bicomponent fibers. The lack of simulation tools capable of predicting these mechanical consequences limits process optimization in industry.

While prior work has extensively covered melt spinning models and component-level material behavior, few have critically examined how the combined viscoelastic properties influence final fiber elasticity in a side-by-side configuration. This study aims to fill that gap by offering a simulation-backed interpretation of the mechanical consequences of mismatched polymer properties (PA6 vs. PA66). This is particularly relevant for industries focused on enhancing product performance in textiles, [7,8,9] filtration, and composites, where fiber elasticity critically influences product durability and comfort.

This study addresses that gap by developing a validated 2D simulation model for PA6/PA66 bicomponent spinning using ANSYS Polyflow 2024 R2. The model couples viscoelastic flow with non-isothermal crystallization behavior to explain reduced elasticity and offers guidelines for improving it through process control.

Bicomponent fibers with exceptional performance can be divided into numerous groups based on how the two polymers are arranged in the cross-section [10]. Crimped fibers were first created by exploiting the difference crimp shrinkage of two polymers, and parallel bicomponent fibers were produced. The core/sheath structure was widely used in the manufacture of bicomponent fibers because it allowed for the inclusion of the desirable properties of both the core/sheath components. INS’s bicomponent fibers are made up of a sea and islands polymer studied. By removing the marine polymer, these fibers can acquire a superfine structure [11]. The process of separating hollow pie-wedge bicomponent fibers into superfine fibers can be accomplished via mechanical, thermal, or chemical treatment [12,13,14]. Frendenberg’s Evolon, made of 16-pie bicomponent fibers, is a well-known example of a commercial pie wedge superfine nonwoven material [15,16,17].

Previous [18] research on bicomponent materials primarily focused on exploring the mechanical properties and various techniques associated with them. Hollowell et al. [19] employed a hollow pie wedge bicomponent in their study, using the hydro-entangling method to create nonwoven fabrics characterized by reduced porosity and enhanced tear strength [20,21].

In a recent investigation conducted by Lu and Qian [22,23], the authors delved into the technique of splitting bicomponent new spun-bond fibers and concluded that hydro-entangling yielded the most favorable outcomes. Yeom and Pourdeyhimi [24] examined the feasibility of utilizing INS spun-bond webs for aerosol filtration and found that these webs exhibited high filtration efficiency. Notably, the filtration patterns in fibrous media revealed that particles within the 0.2 to 0.3 mm range experienced the highest penetration.

Fedorova and Pourdeyhimi [25] described the INS fiber manufacturing process, emphasizing how the marine polymer component can fibrillate, thereby releasing the island structures and enabling the formation of sub-micron fibers. In another study by Zhao and Liu [26], they manufactured PET/PA6 hollow pie wedge bicomponent fibers and observed that the drawing parameters significantly influenced the fineness and strength of the fibers.

Gong and Nikoukhesal [27] conducted research in which they produced microfiber nonwoven fabrics using pie wedge bicomponent methods such as air-laying and hydro-entanglement. Their findings indicated that these microfiber nonwoven fabrics outperformed homocomponent counterparts in terms of tensile strength, elongation, and water absorption. The manufacturing process of fibers, particularly the drawing process, plays a pivotal role in shaping their characteristics and attributes.

Above of all, these studies focus on one component behavior and do not discuss viscoelastic analysis of bicomponent melt spinning towards single filaments, so we chose this material. The elasticity of PA6/PA66 bicomponent melt spinning refers to the ability of a bicomponent fiber to stretch and recover its original shape after being subjected to a force. It is an important property of fibers used in various applications, including textiles, composites, and nonwovens. We integrate a bicomponent melt spinning 2D model by using Polyflow [28] and verify it by modelling the Bheda and Spruiell [29] melt spinning process and predicted temperature and other spinning variables with free surfaces by experimental observations to validate the model [30]. This research investigates the use of Polyflow simulations to determine why the elasticity of PA6/PA66 bicomponent fibers is lower, as shown by experiments, and we explain the why this occurs during the melt spinning process. This research explores the different aspects of validation against experimental data, sensitivity analysis, and process optimization. By using a methodical approach, we hope to identify the key concepts and strategies that successfully increase fiber elasticity, paving the way for the creation of superior materials with a wide range of industrial uses [31].

## 2. Establishment of Melt Spinning Mathematical 2D Model

**Figure 1:**
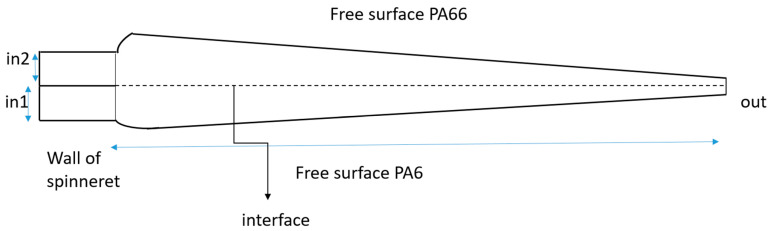
Simulated 2D geometry of the PA6/PA66 bi-component spinning process.

The simulation of the PA6/PA66 side-by-side bicomponent melt spinning process (Figure 1) primarily focuses on melt flow through the spinneret orifice, the conversion of shear stress into tensile tangential stress during extrusion, and the subsequent uniaxial drawing of the filament. The entire sequence—from stretching to fiber solidification—is modeled and analyzed, with emphasis on the constitutive and crystallization equations.

### 2.1. Model

Details about the model: In1 and In2 are located on both sides of the orifice, each with a diameter of 0.28 mm and a length of 0.84 mm. The fiber length is 1.5 m. These parameters are based on experimental data provided by Shanghai Textile Research Institute Co., Ltd. (Shanghai, China).

### 2.2. Governing Equations

For the simulation of melt spinning, the flowing melt must satisfy the laws of mass conservation, momentum conservation, and energy conservation [32]. These laws are embodied in fluid mechanics by equations such as the continuity equation, the momentum equation, and the energy equation; this model is also based on these three equations to derive a closed system of equations to describe the melt flow during the melt spinning process. At the same time, the following assumptions are made for the model on the basis of satisfying mass conservation, energy conservation, and momentum conservation [33,34,35].

1. Polymer melt is incompressible.
2. During the spinning process, the fibers are stretched vertically downward.
3. The fiber interface is a standard circular cross-section, and the diameter changes with the spinning process.
4. The melt has no slip on the tube wall, and the wall velocity is 0.
5. The volume flow rate is constant and the non-isothermal flow is steady laminar flow.
6. Ignoring the effects of inertial force and gravity and avoiding neck problems during high speed.
7. The surface convection heat transfer coefficient includes the influence of the radiation heat transfer coefficient.
8. The effects of fluid dynamics and thermal interactions between adjacent fibers are not considered.
9. No in-depth studies on the interface but on mechanical behavior like elasticity.

These assumptions were validated based on Reynolds and Froude number estimates. At a typical industrial take-up velocity of 1000–1500 m/min, the inertial forces (estimated <1% of viscous forces using Re<<1) are negligible. Similarly, the gravitational effects are minor compared to axial drawing forces and pressure gradients. Fiber–fiber interactions are ignored due to isolated single-filament analysis, which aligns with the scope of the current study.

### 2.3. Mesh

**Figure 2:**
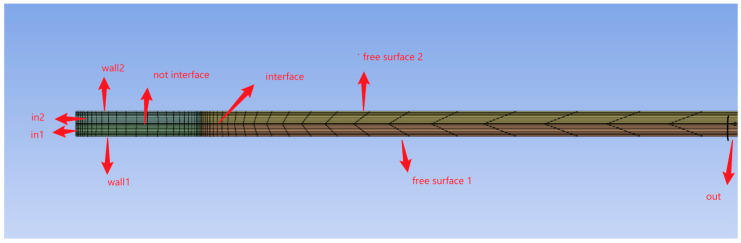
The two-dimensional (2D) mesh model used for simulating the PA6/PA66 bicomponent melt-spinning process [36].

A 2D mesh (Figure 2) was developed for the model, as high-quality meshing is a critical step in achieving accurate simulation results. Computational Fluid Dynamics (CFDs) was selected as the simulation solver. The model comprises two main domains, each containing a subdomain. Nodes: 21,903, elements: 20,840.

Continuity:

(1) $$\;Wi=\pi R^{2}\rho iVi\;\;\;\;\;\;\;\;\;\;\;\;\;\;\;i=1,2$$

Momentum equation:

(2) $$W\left(\frac{dV}{dy}\right)=\frac{d}{dy}\left(A\left(\tau_{yy}-\tau_{xx}\right)\right)-\rho_{a}C_{d}R\pi {\left(V-V_{a}\right)}^{2}+A\rho g+\pi \sigma \frac{dR}{dy}$$

The averaged momentum balance equation, neglecting surface tension effects under steady-state conditions is [36] (see Vassilatos et al., 1992 [30]).

The mean tensile force at a given point y along both side of fibers. The second element represents the air drag force. The quench air viscosity, denoted as “a,” is a crucial component. Finally, the symbol “g” represents the acceleration due to gravity. The equation provided represents the force of gravity acting on a fiber, where A = π. R^2^ represents the cross-sectional area of the fibers and s represents the surface tension of the filament.

Energy equation:

(3) $$\rho iC_{pi}\left(V\frac{dT}{dy}\right)=\left(\tau_{yy}-\tau_{xx}\right)\frac{dV}{dy}-\frac{2h}{R}\left(T-T_{a}\right)+\rho i\Delta H_{fi}V\frac{dX}{dy}\;\;\;\;\;\;\;\;\;\;i=PA6,PA66\;\;\;\;\;$$

Equation (3) defines C_p_ as the overall system’s heat capacity, which is typically affected by temperature and crystallinity. On the RHS, the first term is the convective heat transfer term between the filament and quench air on both sides of filament one is PA6 and another is PA66, where h is the coefficient. Viscous dissipation is the second phase.

Hf is the crystallization heat per unit, while the last term represents latent heat release. At the systems average absolute degree of crystallinity (mass percentage of crystals), mass and axial position z, Ziabicki et al. [37] employed the energy equation degree of transformation, while our approach matches Zieminski and Spruiell [6]. Without crystallization, the last term on RHS of Equation (3) disappears. Radiation effects and axial conduction follow Denn [5] and were disregarded in the energy equation.

Boundary conditions:

In1 = In2: (MFR) w = 0.67 g/min, *T*_1_ = 533, T_2_ = 553 K,

Wall: *T*_1*wall*_ = 533 K, *T*_2*wall*_ = 556 x = *V*y = 0,

Free surface both sides: *V*_x_ = 0, *τ*_x_ = 0, α(*T*−*Tair*),

Out: y = 1000 m/min, Δ*q* = 0, and

Interface:

**n. v** = 0 (4)

The condition “n. v = 0” is often referred to as the “no-slip” condition. This means that at the interface between the two components of the bicomponent fiber, the fluid or polymer does not slip along the boundary. In other words, the velocity of the fluid or polymer at the interface is equal to zero, and it adheres to the surface of the fiber.

Heat exchange equation:

The heat transfer coefficient is related to the fiber speed and the blowing speed. The convection heat from the air to the fiber surface can be expressed, according to Dawei [38,39], in this equation using the user define function (UDF).

Δ*q* = (*T*−*Ta*ir) (5)

(6) $$\alpha =9.287\times 10^{-5}{\left(\frac{V}{A}\right)}^{0.287}=181.07(V{)}^{0.574}$$

(7) $$\alpha =181.07(V{)}^{0.574}{\left[1+{\left(\frac{8V_{\mathrm{air}\;}}{V}\right)}^{2}\right]}^{0.287}$$

The V_air_ velocity of air, V_air_ = 0.45 m/s.

The Arrhenius law:

(8) $$H\left(T\right)=\exp\left[\alpha \left(\frac{1}{T-T_{0}}-\frac{1}{T_{\alpha }-T_{0}}\right)\right]$$

where the activation energy is α, and Tα is a reference temperature at which H(T) equals 1 and α signifies the energy of activation.

A mesh independence study was conducted by comparing velocity and stress profiles at different node densities (130,000, 210,000, and 300,000 elements). Less than 2% variation was observed between 210,000 and 300,000 meshes, confirming convergence. Therefore, the 21,903-node mesh was used for all simulations.

### 2.4. Constitutive Equation

Constitutive equations used to describe polymer melt flow are generally categorized into two types: generalized Newtonian models and viscoelastic models. Given that the primary objective of this study is to analyze melt extrusion behavior through the fine pores of a spinneret, the Phan–Thien–Tanner (PTT) difference viscoelastic model is deemed most appropriate for this application [40,41]. This model effectively captures the shear-thinning behavior observed at high shear rates, as well as the degradation of normal stress components. In this context, the additional stress tensor T**T** is defined as follows:

(9) $$T=T_{1}+T_{2}$$

(10) $$\exp\left[\frac{\varepsilon \lambda }{\eta_{1}}t_{r}\left(T_{1}\right)\right]T_{1}+\lambda \left[\left(1-\frac{x_{i}}{2}\right)T_{1up}+\frac{x_{i}}{2}T_{1\;\mathrm{low}\;}\right]=2\eta_{1}D$$

(11) $$T_{2}=2\eta_{2}D$$

In the formula, *η*1 is the specified viscosity coefficient, *η*1 = (1 − *r*atio) *η* *η*2 refers to the Newtonian component viscosity coefficient, *η*2 = *ra*ti*o*∙*η*, *λ* represents the relaxation time, *tr* represents the tensor trace, and D represents the deformation rate tensor.

Parameters for PA6 and PA66 (e.g., ε, ξ, and λ) were obtained from the literature [4,41] and matched with rheological measurements conducted in our lab using a rotational rheometer (Model MCR 302, Anton Paar GmbH, Graz, Austria) at 553 K. For example, ε = 0.008 (PA6) and ε = 0.001 (PA66) are consistent with their shear thinning behavior under high-speed melt spinning conditions.

### 2.5. The Kinetics Crystallization Model

The kinetics crystallization model employed in this study was based on the work of Nakamura, Lauritzen, Hoffman, and Spruiell [42,43,44].

During the melt spinning process, when polymer melt is extruded through the spinning hole, it will undergo a cooling and crystallization process.

The polymer molecules will transform from na amorphous to crystalline aggregated structure.

(12) $$\;\begin{array}{c} R_{ate}=\vartheta_{\infty }\left(1-\vartheta {/}_{\vartheta_{\infty }}\right)K_{max}exp\left[-4ln2{\left(\frac{T-T_{max}}{W}\right)}^{2}\right.\\ \left.+\frac{C}{{\left(\Delta \alpha_{i}\right)}^{2}}C_{op}^{2}{\left(\tau_{yy}-\tau_{xx}\right)}^{2}\right]\;\;\;\;\;\;\;\;\;\;\;\;\;\;\;\;\;\;\;\;\;\;\; \end{array}$$

In the formula, T_m_ is the melting temperature, T represents the temperature, *ϑ*∞ represents the maximum crystallinity, *Tma*∞ represents the maximum crystallization rate temperature, *Kma*igh represents the crystallization rate at a temperature of *T*_*ma*x_, and *W* represents the half-crystalline width.

The induction coefficient, *Cop*, represents the stress optical coefficient and *τ*yy−*τ*xx represents the first normal stress difference.

### 2.6. Spinning Process Parameters

**Figure 3:**
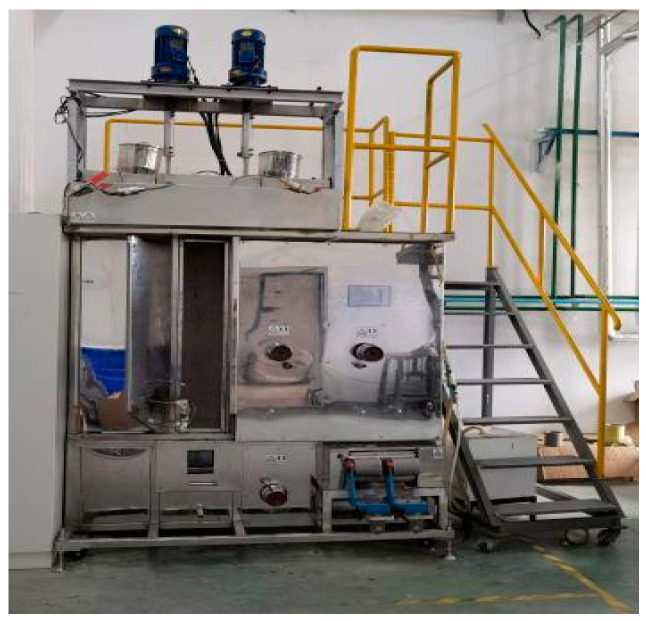
Industrial bicomponent spinning equipment.

A low-speed bicomponent melt spinning machine was employed for the spinning process. The raw materials for each component were fed separately into hoppers positioned on either side of the machine. After passing through the screws, the melt ratios of the two polymers were regulated by a metering pump, which directed the melt mixture to the spinning assembly. The resulting filament bundle was extruded through the spinneret, passed through the drafting rollers after oiling, and finally wound onto a silk reel to complete the spinning process. The detailed parameters of the spinning components are provided in Table 1, while the spinning equipment and its schematic layout are illustrated in Figure 3. The melt spinning and drafting process parameters are summarized in Table 1 and Table 2.

The extruder used had a screw speed of 80 rpm, with a screw L/D ratio of 30:1. The feed rate was maintained at 10 g/min per component. A temperature gradient was applied from 493 K at the hopper to 553 K at the spinneret. These conditions match standard industrial configurations and were validated by our experimental collaborators.

### 2.7. Material Data

Table 2 shows the material properties from the experimental data [2,3,45,46] and some values calculated based on the model.

### 2.8. Validation of the Model

**Figure 4:**
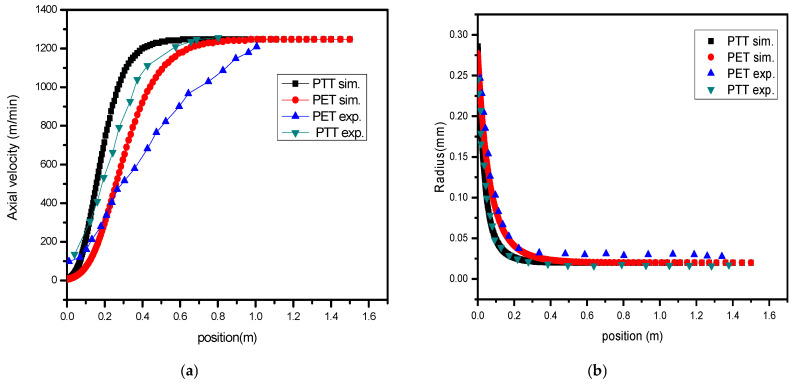
The data show (**a**) the axial velocity and (**b**) the radius of the studied PET and PTT to validate the model.

Two sets of data were used to confirm the model: (i) data from Gregory [47], for spinning PET and PTT [48] fibers; and (ii) data from our own experiments for spinning PA6 and PA66 blends. We used published data on PET and PTT for the above model and the velocity and radius results are shown in Figure 4a,b.

Figure 4 shows model validation performed using both the literature data (PET/PTT from Gregory [47] and Zhang [48]) and our own experimental results for PA6/PA66 spinning. The Root Mean Square Error (RMSE) between the predicted and observed velocity profiles was 7.8%, and 5.1% for the radius. These values fall within the acceptable simulation accuracy for industrial fiber modeling.

## 3. Results and Discussion

### 3.1. Pressure

**Figure 5:**
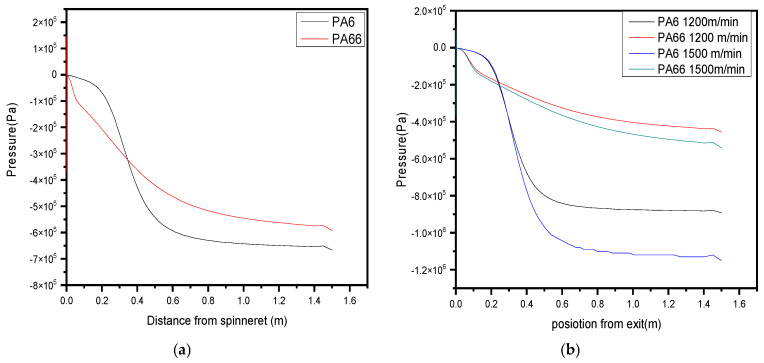
(**a**,**b**) The predicted pressure drop after exit to spinneret along the spin line of the bicomponent with different speeds.

Different pressure drop curves for PA6 and PA66 should normally be visible in a bicomponent simulation. Compared to PA6, PA66 frequently exhibits different drop trends in simulations as shown in Figure 1, with steeper pressure drop curves due to its greater melt viscosity. This distinction is particularly apparent when the materials undergo the same processing conditions. PA66 typically has a higher melt viscosity than PA6, which makes it less elastic. Dominance of PA66 in the bicomponent blend can lead to lower elasticity and, consequently, a low-elasticity pressure drop trend. A greater pressure drop, as shown in Figure 5a, leads to greater flow resistance in the fiber. This increased pressure drop may be a sign of decreased elasticity and increased internal friction in the fiber. This might also imply that the fiber is less prone to distortion under stress and is stiffer overall. The pressure drop trends for PA6 and PA66 in a bicomponent simulation should normally exhibit variations in their respective pressure drop curves’ slopes, peaks, and general forms. Compared to PA6, PA66 may have a steeper and more noticeable pressure drop curve due to its higher melt viscosity.

Variations in viscosity and flow resistance significantly influence the observed pressure drop. Generally, a higher pressure drop indicates increased viscosity and reduced elasticity in the fiber. Elasticity, by definition, refers to a material’s ability to return to its original shape after deformation. Materials with lower elasticity tend to have higher pressure drops, reflecting their limited ability to recover after stretching. Additionally, at the interface where the two polymer components of the bicomponent fiber meet, a transition zone may form. This region can appear as a noticeable feature on the pressure drop graph, often presenting as a peak or a change in slope, as illustrated in Figure 5a.

Although they are both polyamides, PA6 and PA66 have distinct material characteristics. Generally speaking, PA6 is more elastic and has a lower melting point than PA66. Because PA6 has a lower glass transition temperature (Tg), it showed less flexibility in studies by [49] but not in numerically validated models. A bicomponent composite in which PA66 predominates can experience a substantial reduction in its overall elasticity. A transition zone may form at the interface where the two components merge during bicomponent extrusion. This zone can influence the pressure drop and may appear as a noticeable feature on the pressure drop curve.

As shown in Figure 5a, the simulation matches the trend observed by Sun et al. [4], where PA66 exhibits a higher pressure drop due to its higher viscosity. This drop is correlated with decreased chain mobility and thus lower elasticity, consistent with findings by Doufas et al. [50].

An increase in take-up velocity may cause the pressure drop to rise more noticeably and steeply. This is due to the fact that higher extrusion rates are required due to the greater take-up velocity as shown in Figure 5b higher extrusion rates, also resulting in higher shear and flow rates, which may raise pressure drops. On the graph, the trend could appear to be steeply sloped or exponential.

An increase in pressure drop typically signifies higher viscosity and internal friction, conditions that are generally associated with reduced elasticity. Conversely, materials with lower viscosity and internal resistance tend to exhibit lower pressure drops, which correlate with improved elasticity. Simulation results suggest that a rapid rise in take-up velocity may lead to flow instabilities and turbulence during the extrusion process. These disturbances can produce irregular pressure variations, often reflected as sharp or erratic fluctuations in the pressure drop curve. For instance, a sudden transition from low to high take-up velocity may trigger an abrupt spike in pressure due to the rapid shift in flow dynamics.

### 3.2. Prediction of Differences in the Stress Component Along the Fiber

**Figure 6:**
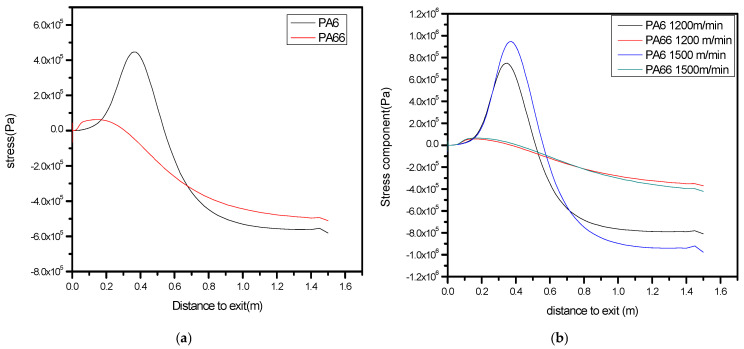
(**a**,**b**) The predicted stress components along the spin line of the bicomponent with different speeds.

The length of the fiber along the spin line is shown on the graph’s X-axis. It usually begins where the polymers are initially extruded via the spinneret and continues until the fiber is gathered or wound onto a spool. The magnitude of the stress component at various points along the spin line is represented by the Y-axis. PA66 may be subjected to noticeably greater stresses than the PA6 under the same process conditions when there is an unequal distribution as shown in Figure 6a. As a result, there is a discernible peak difference, with the stress peak of one component being significantly higher than the other.

Uneven stress distribution can lead to low elasticity. High, uneven stresses can cause strain localization, where one component deforms irreversibly and does not return to its original shape when the stress is removed.

The fiber’s elasticity can be adversely affected when the axial stress distribution consistently shows elevated stress levels along the spin line. In such cases, the fiber may lose its ability to return to its original shape after being stretched or deformed. As illustrated in Figure 6a, noticeable differences in stress distribution between the two polymer components (PA6 and PA66) can be observed in a bicomponent fiber. At certain points along the spin line, one polymer may experience greater stress than the other, likely due to differences in mechanical properties and how the materials are arranged within the fiber’s cross-sectional structure.

In a bicomponent fiber with varying axial velocities, regions with higher velocities may experience more internal stress. This can lead to localized weaknesses in the fiber, contributing to low elasticity and potentially affecting overall mechanical properties as shown in Figure 6b if take-up velocity increases. The axial stress also shows variation in fiber and shows a high peak for PA66 and PA6, respectively; accordingly, this fiber will more stiff if take-up velocity increases. The areas of the fiber cross-section where the stress component is most noticeable are shown by the highest point or points on the graph. Areas of high stress are more likely to have an impact on characteristics like elasticity and strength. For polyamides (PA6 and PA66), an uneven distribution of stress—where one component experiences significantly higher stress—can result in permanent deformation and reduced elasticity. Conversely, a more uniform stress distribution supports elastic deformation and enhances the overall elasticity of the final fiber.

### 3.3. Tensile Stress (Force/Area) Differences in the Bicomponent Fiber

**Figure 7:**
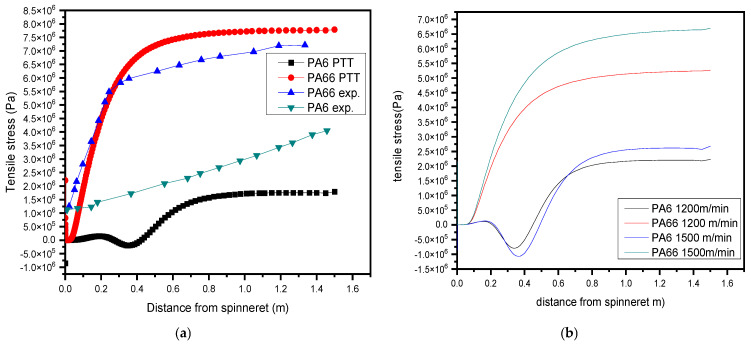
(**a**) A comparison of the axial tensile stress with low speed [2,3]; (**b**) fiber axial tensile stress with variable speeds.

As shown in Figure 7a, the tension stress is not very high at the start of the spin line because polymer melt has just begun to leave the spinneret. This is the first step in the fiber forming process. As the fiber undergoes the process of being pulled and stretched along the spin line, there is a progressive increase in tensile stress. The observed rise in magnitude is linked to the mechanical forces exerted on the polymer, which results in the orientation and alignment of the polymer chains, ultimately leading to the formation of the fiber. The observed behavior of the material at this phase exhibits a highly elastic response, characterized by its ability to undergo stretching without experiencing substantial irreversible deformation, which is explained by Shin et al. [51].

The primary factor contributing to the low elasticity found in the simulation findings is the inherent stiffness and reduced elasticity of PA66. The blended fiber’s overall elasticity is diminished when the PA66 exhibits dominance at different stages of spinning and drawing. One important effect is the change while spinning from the more elastic PA6 to the stiffer PA66. Following this transition, the fiber undergoes reduced recoverability, indicating that it does not fully go back to its initial condition following deformation. This observed behavior is consistent with the characteristic pattern seen on a tensile graph, wherein a notable decline in slope is often observed following the attainment of the yield point.

It can be observed that the tensile stress is comparatively lower for PA6 and greater for PA66 at every position along the spin line. A higher tensile stress for PA66 means that stretching it will take more force and that it will reach its yield point faster, giving way to plastic deformation. The lower tensile stress exhibited by PA6 suggests that it possesses a higher degree of stretch ability, allowing it to undergo elongation with less stress accumulation, hence demonstrating its elastic characteristics. But there is a downward trend at the phase trans position, and it also increases with increased winding speeds as shown in Figure 7b. The observed phenomenon can be attributed to the inherent disparities in the mechanical characteristics of the materials in question as discussed by F. Rybnikar and colleagues [52].

The primary factor contributing to the low elasticity found in the simulation findings is the inherent stiffness and reduced elasticity of PA66. The overall elasticity of the composite fiber is diminished when the dominance of PA66 is observed at different stages during the spinning process. The lower tensile stress exhibited by PA6 suggests a higher degree of elasticity, whereas the higher tensile stress observed in PA66 shows its increased stiffness and less elasticity. The reduction in elasticity observed in the simulation can be linked to the material transition from the more flexible PA6 to the stiffer PA66 during the spinning process. This behavior corresponds with the trends reported by J. Sun and Dong [4,50] in their tensile stress analyses. The shift from an elastic to a more rigid phase contributes to the diminished elasticity, a pattern that is also evident in standard tensile stress–strain curves, as illustrated in the experimental graph.

### 3.4. Temperature

**Figure 8:**
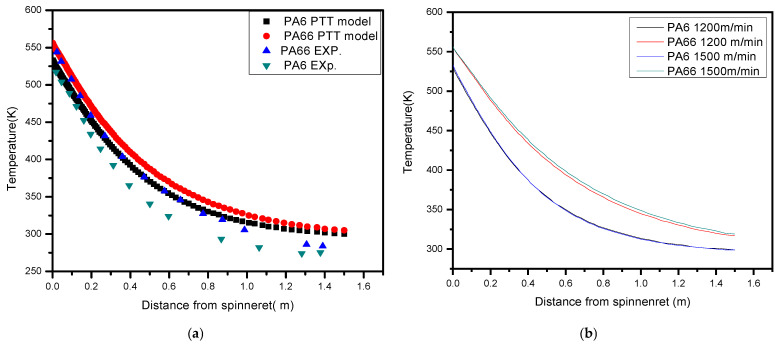
(**a**) A comparison of the temperature with low speed [2,3]; (**b**) fiber temperature with different speeds.

The X-axis represents the distance from the spinneret, illustrating how far the fibers have moved away from the point of extrusion. Typically, this distance increases as the fibers move away from the spinneret. The Y-axis shows the temperature, which is given in degrees Kelvin. It shows how hot the fibers are at different places along the spin line towards take-up fiber position as shown in Figure 8a. The temperature is usually highest near the spinneret, where the molten PA6 and PA66 polymers are extruded. Both polymers are now semi-molten and getting near to melting temperatures. In close proximity to the spinneret, where elevated temperatures prevail, the partially molten polymers exhibit enhanced intermolecular adhesion. This phenomenon has the potential to provide a robust and cohesive first connection between the two constituent polymer elements. Strong initial bonding can make the fiber more flexible by making sure that the PA6 and PA66 stages hold together well as shown in the studies of [4,50].

The fibers start to cool down as they move away from the spinneret and come into contact with air or a cooling medium. Each fiber loses heat, so the temperature slowly drops along the spin line. As shown in the graphs in Figure 8a,b, the fibers cool down and move away from the spinneret, and the temperature drops, which helps PA6 and PA66 separate into different phases. To keep the unique side-by-side shape and let both polymers add to the fiber’s mechanical properties, phase separation is very important. Phase separation is a key factor in the success of PA6/PA66 bicomponent melt spinning [51].

The difference in temperature between PA6 and PA66 in the melt spinning procedure impacts the extent of intermolecular bonding at their contact. One polymer might be more molten than the other when there is a noticeable temperature difference as shown in Figure 4a experimental and simulation results. The interaction between the two polymers has the same structure at different levels of bonding. Enhancing the mechanical strength and flexibility of fibers can be performed by stronger bonding, which is often facilitated by a lower temperature difference. Figure 8b shows take-up speed increases under the same spinning conditions, PA6 is impacted little compared with PA66. With same spinning process, the difference after the blending stage increases until solidification is reached.

### 3.5. Axial Velocity

**Figure 9:**
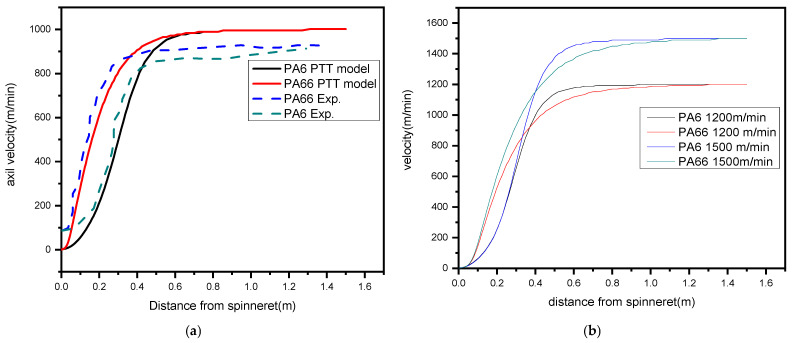
(**a**) A comparison of the axial velocity with low speed [2,3]; (**b**) the predicted axial velocity of different speeds.

Figure 9a shows the simulated and experimental data. The Y-axis represents the axial velocity of the polymer in units like meters per min (m/min) or any other relevant speed unit. This is a measure of how fast the polymer filaments are being drawn or pulled along the length of the spinning process. The X-axis represents the position or length along the spinning process. It usually starts at the spinneret and progresses along the length of fiber formation.

Usually, there is a significant spike in axial velocity at the spinneret at the beginning of the graph. This happens because the polymer filaments accelerate very quickly when they leave the spinneret. The stark difference in axial velocity can result, as shown in graph Fig-a, in a non-uniform fiber structure, with regions of high and low elasticity. The overall fiber may exhibit a variable and less predictable elasticity profile.

A significant difference in axial velocity between PA6 and PA66 on the graph indicates that the two polymers are being elongated at different rates. This disparity leads to uneven stretching and inconsistent alignment of the polymer chains within the fiber. As supported by studies [4,50], such velocity variations contribute to irregular molecular orientation and fiber thickness. These inconsistencies negatively impact the fiber’s elasticity by reducing its ability to deform and return to its original shape. In bicomponent fibers, regions with differing axial velocities often experience uneven internal stresses, with faster-moving areas subjected to greater stress levels. The occurrence of localized weaknesses in the fiber might result in less elasticity and potentially impact the overall mechanical characteristics [4,50,51]. Phase separation between the PA6 and PA66 components can be affected by the velocity difference. Better phase separation may result in discrete areas with unique characteristics that have varying effects on elasticity with et al. studies [52].

The resultant fibers’ elasticity may be deliberately controlled by varying the axial velocity difference in a bicomponent melt spinning line. The two components may have different levels of elasticity as a result of an increase in the axial velocity difference, which would make the fiber less homogeneous. On the other hand, a smaller axial velocity difference encourages a more homogeneous fiber structure and balanced elasticity.

Figure 9b shows that the polymer crystallization process may be impacted by the decreased cooling time resulting from the higher take-up velocity. By increasing the winding speed, the duration of fiber exposure to different stages of the spinning process, such as the draw zone, is reduced. The duration of residence is decreased. Increased amorphous areas in the fibers due to a higher cooling process might affect the structural characteristics of the material. Reducing the axial velocity difference means that the two materials, PA6 and PA66, are drawn more evenly, which results in a balanced distribution of axial velocity along the spin line. Better phase separation between PA6 and PA66 borders may be seen in the components. This produces more consistent areas with distinct mechanical characteristics, which is beneficial for controlling elasticity.

The difference in axial velocity along the spin line during the melt spinning process plays a crucial role in determining the properties of bicomponent fibers. An increase in this velocity difference tends to affect the PA66 component more significantly due to its higher stiffness. As shown in Figure 9b, a rise in winding speed results in reduced axial velocity on the PA66 side, while the PA6 side exhibits a comparatively increasing trend along the spin line. These variations influence the overall fiber elasticity, as also demonstrated in previous studies [48]. In contrast, reducing the axial velocity difference leads to a more uniform and balanced distribution of mechanical properties, including improved elasticity [51].

### 3.6. Radius

**Figure 10:**
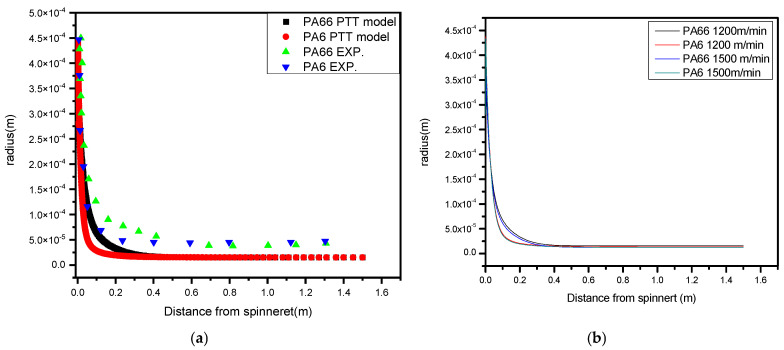
(**a**) A comparison of the radius with low speed [2,3]; (**b**) fiber radius with different speeds.

Figure 10a illustrates that the model and experimental data profiles followed a similar pattern. As stated by the momentum equation (Equation (2)), rheological forces are responsible for the observed fluctuations in fiber diameter. Near the spinneret, the fiber experiences both strong acceleration and attenuation due to rheological pressures. Due to viscoelasticity, the simulation results did not match the experimental results at high pick up speeds.

The fiber’s performance in a bicomponent melt spinning process using side-by-side combinations of PA6 and PA66 can be significantly influenced by simulations comparing experimental findings for fiber radius. As shown in Figure 10a, the PA6 and PA66 polymers are extruded side by side through a spinneret at the start of the melt spinning process. At first, the resulting bicomponent fiber has a constant diameter along its length. Usually, the simulation results will indicate a decreasing trend in fiber radius as proceeding along the spinning line. This indicates the fiber’s thinning and stretching, particularly in the PA6 areas, are more elastic. The sections of the fiber that are thinner, which are frequently associated with the PA6 side, have more elasticity. PA6 may stretch more easily due to its molecular structure, and thinner sections have less substance, making them easier to stretch without permanent deformation. The radius of the fiber is a critical factor in influencing its elasticity, but in bicomponent simulated results, due to a higher winding speed, as shown in Figure 10b, the fiber diameter tends to decrease. This is because a higher winding speed results in a higher draw ratio, which stretches the fibers and reduces their diameter. Consequently, the graph will likely show a decreasing trend in fiber diameter as winding speed increases. In a bicomponent fiber configuration, such as the PA6/PA66 combination, the polymer distribution of each component has a significant role in determining the radius of either side of the fiber. The distribution of the polymers along the fiber cross-section may be impacted by an increase in winding speed, which might result in a little variation in radius between the fiber’s two sides.

Because of its higher elasticity and susceptibility to stretching, the side with more PA6 may show a somewhat larger diameter loss if the distribution of PA66 and PA6 is not exactly even across the cross-section. A slight disparity in radius may arise between the two sides [4,50].

The distribution of each polymer type can affect the radius on either side of a bicomponent fiber that is side by side, like PA6/PA66. The distribution of polymers throughout the cross-section of a fiber may be influenced by an increase in winding speed, which might result in a little disparity in radius between the two sides of the fiber [53].

Due to its greater elasticity and susceptibility to stretching, the region with a higher concentration of PA6 may exhibit a slightly more pronounced reduction in diameter, especially if the PA6 and PA66 components are unevenly distributed across the fiber’s cross-section. This uneven distribution can lead to a minor difference in radius between the two sides. An increase in the radius disparity along the spin line between the PA6 and PA66 sides suggests a relative thickening on one side of the fiber compared to the other.

This increase in the difference in radius may have a major effect on elasticity. The side exhibiting a greater radius (referred to as the thicker side) is expected to have diminished levels of stretchability and elasticity in comparison to the thinner side (characterized by a lower radius). Due to the thicker side’s increased resistance to stretching, the fiber’s overall elasticity may be decreased [54].

### 3.7. Crystallinity [2,3]

**Figure 11:**
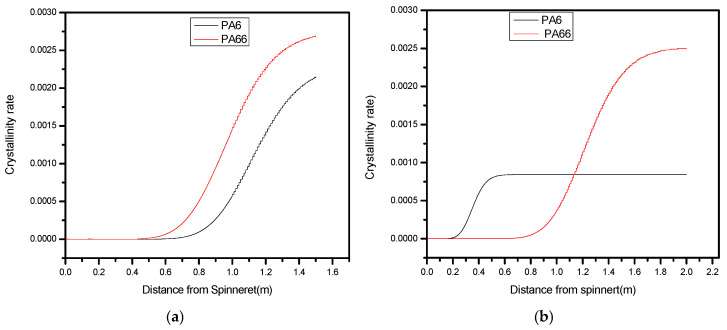
(**a**,**b**) The same processing condition while increasing winding speed from 1000 m/min to 1200 m/min.

In Figure 11a, the Y-axis shows the rate of crystallinity, while the X-axis shows the length from spinneret. The crystallinity graph peaks or is high near the spinneret exit in the context of melt spinning; according to experimental data, this indicates that the polymer filament is undergoing significant crystallization as it exits the spinneret [4]. The region near the spinneret exit is where the freshly extruded polymer filament comes into contact with the surrounding air or a cooling medium. This rapid cooling can promote the nucleation and growth of crystalline regions within the polymer.

In a side-by-side arrangement, the crystallinity of PA6 and PA66 might not be evenly balanced. When one component exhibits a notably higher degree of crystallinity compared to the other, this might lead to less elasticity. Antonios K. Doufas [50] studied and explained how this impacts crystallization on final fibers. Reduced elasticity may be explained by looking at the crystallinity rate difference, as shown in Figure 11 comparing PA66 and PA6 fibers.

Figure 11 was based on simulated crystallinity using the Nakamura model. Validation was achieved by comparing simulated values with differential scanning calorimetry (DSC) data obtained from lab-prepared fibers (±5% deviation in peak crystallinity).

#### Prediction of Crystallites Rate Along the Spin Line

Greater rigidity and decreased elasticity are the results of higher crystallinity, as demonstrated by the PA66 component. The material is less elastic because crystalline areas prevent the polymer chains from stretching and recovering. Because PA6 has more amorphous areas that allow for higher flexibility and stretch ability, its reduced crystallinity enhances its elasticity, which was explained with high speeds studied by J. Sun et al. and Dond and colleagues [4,51].

In Figure 11b, the extruded polymer filament’s rate of cooling is affected by winding speed. Generally speaking, higher drawing and cooling of the filament are caused by greater winding speeds. The amount of time that polymer chains have to organize themselves into crystalline structures may be reduced by faster cooling. Therefore, both PA6 and PA66 components are more likely to have poorer crystallinity when winding speed is increased according to J. Sun et al. [4].

The material’s elasticity tends to be better balanced between the PA6 and PA66 segments when both components have similar and lower crystallinity as a result of the faster cooling brought on by greater winding speeds.

## 4. Conclusions

This study presents a validated two-dimensional (2D) Polyflow-based simulation of the side-by-side PA6/PA66 bicomponent melt spinning process, focusing on the viscoelastic behavior and its effect on fiber elasticity. By coupling the Phan–Thien–Tanner (PTT) viscoelastic model with non-isothermal crystallization kinetics, the simulation accurately predicts key process variables such as pressure drop, axial stress, temperature, velocity, fiber radius, and crystallinity profiles along the spin line.

The results reveal that the PA66 component, due to its higher melt viscosity and crystallization tendency, dominates the stress and structural development within the fiber, leading to reduced overall elasticity. Elevated pressure drops and uneven tensile stress distributions correlate with greater stiffness and lower elastic recovery in the final product. In contrast, PA6 contributes to improved elasticity due to its lower viscosity and greater amorphous content. Simulated predictions for fiber radius and velocity show strong agreement with experimental data, confirming the model’s accuracy and practical relevance.

Importantly, this work identifies critical parameters—such as polymer ratio, take-up velocity, and temperature gradient—that can be strategically adjusted to improve elasticity in industrial fiber manufacturing. This study offers valuable guidance for process engineers and material scientists seeking to optimize bicomponent fiber properties without resorting to time-consuming and costly full-scale trials.

While the current model captures key aspects of mechanical behavior, it does not explicitly account for interfacial phenomena between PA6 and PA66. Future work should incorporate interfacial adhesion modeling and transition to full 3D simulations to capture more complex flow behavior and cross-sectional effects. Such advancements will enable broader applicability of the simulation framework to industrial-scale production of elastic, high-performance fibers.

## Figures and Tables

**Table 1:** Spinning Component Parameters.

PA6	PA66	Spinneret Orifice No.	(Dia × Length of Orifice) mm
533 K temperature	556 K temperature	36	0.28 × 0.84
Winding speed: 1000 m/min	Winding speed: 1000 m/min		

**Table 2:** Material properties of PA6 and PA66 used in the simulation and experimental validation, including rheological and thermal parameters.

Material Properties	PA6	PA66
Intrinsic viscosity, IV, dl/g	2.40	2.40
Specific heat capacity, $C_{p},J/kgK$	1891	2553
Density, $\rho ,kg/m^{3}$	973	1000
Thermal conductivity, λ,w/Mk	0.20	0.21
Melting temperature, $T_{m},K$	533	556
*λ*(s)	0.03	0.01
*ε*	0.008	0.001
*ξ*	0.175	0.15
Ratio	0.20	0.35

## Data Availability

Data are contained within the article.
